# Primary tumour characteristics and axillary lymph node status in breast cancer

**DOI:** 10.1038/sj.bjc.6690629

**Published:** 1999-08

**Authors:** C Yiangou, S Shousha, H D Sinnett

**Affiliations:** Departments of Breast Surgery and Histopathology, Charing Cross Hospital, London, W6 8RF, UK

**Keywords:** axillary lymph nodes, breast cancer, primary tumour characteristics

## Abstract

This paper examines the correlation between axillary lymph node status and primary tumour characteristics in breast cancer and whether this can be used to select patients for axillary lymphadenectomy. The results are based on a retrospective analysis of 909 patients who underwent axillary dissection in our unit. Axillary lymph nodes containing metastases were found in 406 patients (44.7%), all with invasive carcinomas, but in none of the 37 carcinomas-in-situ. Nodal status was negative in all T1a tumours, but lymph node metastases were present in 16.3% and 35.7% of T1b and T1c tumours respectively. When histological grade was taken into account, positivity for grade I T1b and T1c tumours fell to 13.6% and 26.7% respectively. Lymph node metastases were found in 85% of patients with lymphovascular invasion in their tumours as compared to only 15.4% of those without and in 45.5% of oestrogen and progesterone receptor-positive tumours. When one or both hormone receptors were absent this figure was much higher. It appears that for T1a breast cancers axillary dissection is not necessary, whereas for T1b, T1c and grade I T2 tumours other histopathological parameters should be taken into consideration in deciding who should undergo axillary lymphadenectomy. © 1999 Cancer Research Campaign


*British Journal of Cancer* (1999) **80**(12), 1974–1978
© 1999 Cancer Research Campaign
Article no. bjoc.1999.0629

### Primary tumour characteristics and axillary lymph node status in breast cancer

**C Yiangou[1], S Shousha[2] and HD Sinnett[1]**

Departments of [1]Breast Surgery and [2]Histopathology, Charing Cross Hospital, London W6 8RF, UK

**Summary** This paper examines the correlation between axillary lymph node status and primary tumour characteristics in breast cancer and whether this can be used to select patients for axillary lymphadenectomy. The results are based on a retrospective analysis of 909 patients who underwent axillary dissection in our unit. Axillary lymph nodes containing metastases were found in 406 patients (44.7%), all with invasive carcinomas, but in none of the 37 carcinomas-in-situ. Nodal status was negative in all T1a tumours, but lymph node metastases were present in 16.3% and 35.7% of T1b and T1c tumours respectively. When histological grade was taken into account, positivity for grade I T1b and T1c tumours fell to 13.6% and 26.7% respectively. Lymph node metastases were found in 85% of patients with lymphovascular invasion in their tumours as compared to only 15.4% of those without and in 45.5% of oestrogen and progesterone receptor-positive tumours. When one or both hormone receptors were absent this figure was much higher. It appears that for T1a breast cancers axillary dissection is not necessary, whereas for T1b, T1c and grade I T2 tumours other histopathological parameters should be taken into consideration in deciding who should undergo axillary lymphadenectomy.

**Keywords:** axillary lymph nodes; breast cancer; primary tumour characteristics

Axillary lymph node status is probably the single most important prognostic indicator in breast cancer. Clinical evaluation of the axilla is neither sensitive nor specific enough (Sacre, 1986) and radiological tests including mammography, ultrasound scanning and lymphoscintigraphy have not been useful in the preoperative assessment of the axilla (Bruneton et al, 1986; McLean and Eye, 1986; Lam et al, 1996). Immunolymphoscintigraphy using radiolabelled anti-breast cancer monoclonal antibodies achieved a sensitivity of 86% and specificity of 92% (Tjandra et al, 1986*a*, 1986*b*), but it is unclear as to how accurate magnetic resonance imaging of the axilla is. More recently, it has been reported that positron emission tomography can be useful in detecting nodal metastases with 79% sensitivity and 96% specificity for T1 tumours, but for tumours over 2 cm in diameter, these figures improve to 94% and 100% respectively (Avril et al, 1996).

Surgical removal for histological assessment remains the best and most accurate way to assess nodal status. Although it reduces the incidence of tumour recurrence in the axillary lymph nodes, axillary dissection remains controversial. Some advocate axillary dissection for all patients with invasive breast cancers (Fentiman and Mansel, 1991; Cabanes et al, 1992), whereas others have questioned the need for this procedure in patients with early tumours (Silverstein et al, 1994). The anatomical extent and level of axillary dissection has also been discussed extensively, with some showing preference for complete axillary clearance (Fentiman and Mansel, 1991) and others opting for partial dissection and sampling of four lymph nodes (Steele et al, 1985; Greenall, 1995). The latter approach, however, runs the risk of failing to accurately stage the patient, with error rates as high as 24% (Kissin et al, 1982; Kjaergaard et al, 1985).

The aim of this study was to identify the primary histopathological tumour characteristics which correlate with axillary lymph node metastases and determine whether they can be used to predict axillary lymph node status and as criteria in selecting patients for axillary dissection.

### PATIENTS AND METHODS

This is a retrospective analysis of 909 patients with breast cancer who underwent axillary lymphadenectomy in our unit over an 8-year period, from June 1989 to June 1997. Over 95% of patients with invasive cancer underwent a level 3 axillary clearance, whereas a level 1 axillary dissection was performed in nearly all cases of ductal carcinomas in situ (DCIS).

Axillary lymph node status was correlated with a range of histopathological parameters:

a. Tumour size was the maximum diameter of the invasive cancer measured macroscopically and confirmed microscopically by the histopathologist and categorized using the T value of the TNM system: T1a = 0–5 mm; T1b = 6–10 mm; T1c = 11–20 mm; T2 = 21–50 mm; T3 = > 50 mm
b. Histological type
c. Histological grade using the modified Bloom and Richardson system (Elston and Ellis, 1991)
d. Lymphovascular invasion seen in vessels within the primary tumour on haematoxylin and eosin slides
e. Oestrogen and progesterone receptor status assessed either immunohistochemically (in the second half of the study period) or by a radioimmunoassay (in the first half) and was used to divide patients into four groups:

*Received 14 August 1998*
*Revised 23 February 1999*
*Accepted 24 February 1999*

*Correspondence to:* C Yiangou, Department of Breast Surgery, Charing Cross Hospital, London W6 8RF

**Table 1** Histopathological parameters of tumours in this study

| Parameter | *n* | % |
|---|---|---|
| Histological type | | |
| Invasive ductal | 709 | 78.0 |
| Invasive lobular | 86 | 9.5 |
| Ductal in-situ | 37 | 4.0 |
| Special types | 77 | 8.5 |
| Histological grade | | |
| I | 97 | 11.1 |
| II | 492 | 56.4 |
| III | 283 | 32.5 |
| Tumour size (mm) | | |
| 0–5 | 26 | 3.0 |
| 6–10 | 98 | 11.2 |
| 11–20 | 359 | 41.2 |
| 21–50 | 326 | 37.4 |
| >50 | 63 | 7.2 |
| Lymphovascular invasion | | |
| +ve | 293 | 33.6 |
| –ve | 461 | 52.9 |
| NK | 118 | 13.5 |
| Oestrogen/Progesterone receptors | | |
| +/+ | 352 | 40.4 |
| +/– | 108 | 12.4 |
| –/+ | 37 | 4.2 |
| –/– | 139 | 15.9 |
| NK | 236 | 27.1 |

NK = not known.

**Table 2** Breakdown of lymph node-positive cases according to tumour size and histological grade

| Tumour size (mm) | Grade I – *n* (%) | Grade II – *n* (%) | Grade III – *n* (%) |
|---|---|---|---|
| 0–5 | 0 (0) | 0 (0) | 0 (0) |
| 6–10 | 3 (13.6) | 9 (14.1) | 3 (25) |
| 11–20 | 12 (26.7) | 79 (37.8) | 37 (35.2) |
| 21–50 | 8 (42.1) | 114 (65.9) | 84 (62.7) |
| > 50 | 2 (100) | 28 (90.3) | 26 (86.7) |

- ER +ve/PgR +ve
- ER +ve/PgR –ve
- ER –ve/PgR +ve
- ER –ve/PgR –ve.

### RESULTS

Four hundred and six patients (44.7%) of the total of 909 who had axillary lymphadenectomy had nodal metastases and of these 229 (56.4%) had up to three nodes involved. Table 1 shows a detailed breakdown of the histopathological parameters of the tumours of all the patients included in this study.

Eight hundred and seventy-two (96%) of our patients had invasive breast carcinomas and 77 of these fell into the ‘special types’ category, which included mucinous, medullary and tubular carcinomas or a combination of two types of invasive carcinomas. Histological type and grade and tumour size were recorded for every case in this study. However, in the first 2 years of this period lymphovascular invasion was not recorded and hormone receptors were not measured routinely and, therefore, these parameters are not known for 118 and 236 patients respectively (Table 1).

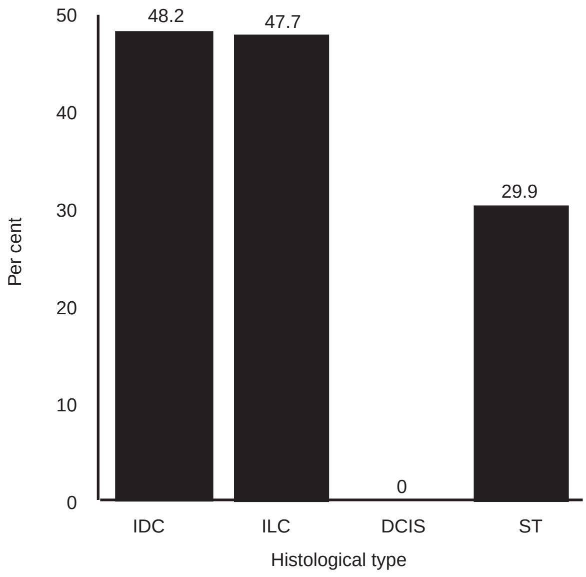

**Figure 1** Axillary lymph node positivity (%) per histological type. ST = special types

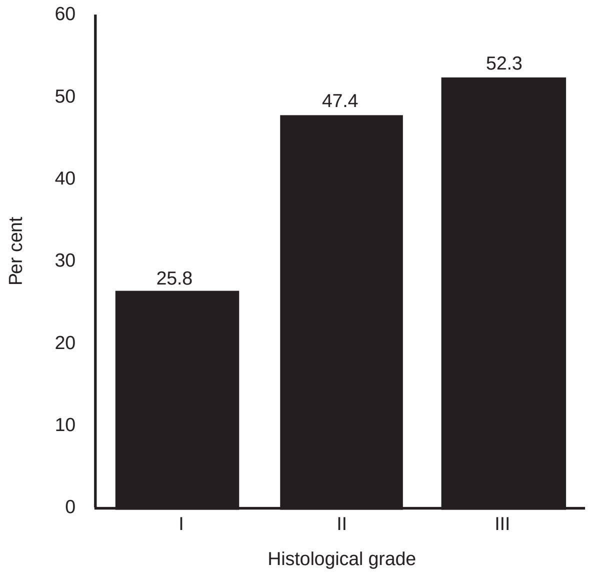

**Figure 2** Axillary lymph node positivity (%) per histological grade

Nearly half of invasive ductal and lobular carcinomas had positive axillary lymph nodes and as expected none of the ductal carcinomas in situ were associated with nodal metastases (Figure 1). Histological grade was related to lymph node status, with only 25% of grade l tumours being associated with nodal metastases as compared to 52.3% of grade III tumours (Figure 2). Tumour size was also another important factor with positivity ranging from 0% in T1a to 88.9% in T3 cancers (Figure 3).

When the last two parameters were combined together (Table 2), for T1b tumours positive axillary lymph nodes were found in around 14% of grade I and grade II, as compared to 25% of grade III tumours. For grade I T1c tumours only 26.7% had metastatic disease in the axilla.

© 1999 Cancer Research Campaign

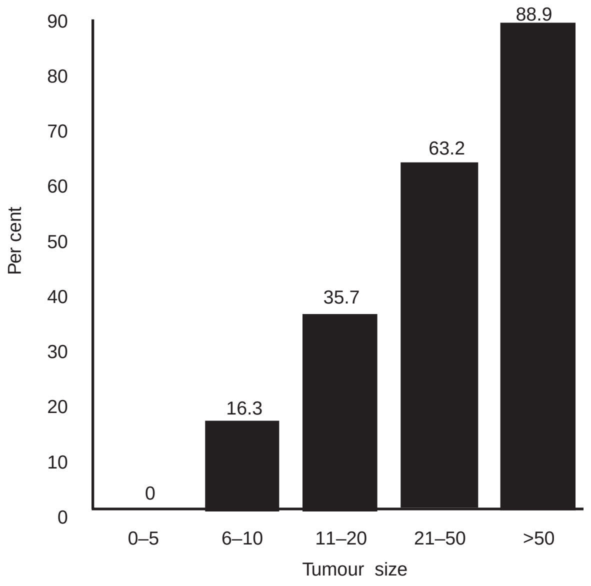

**Figure 3** Axillary lymph node positivity (%) according to tumour size

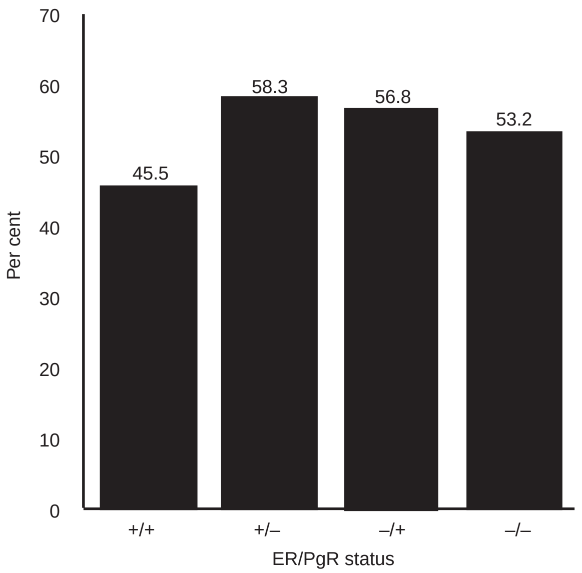

**Figure 5** Axillary lymph node positivity (%) in relation to hormone receptor status

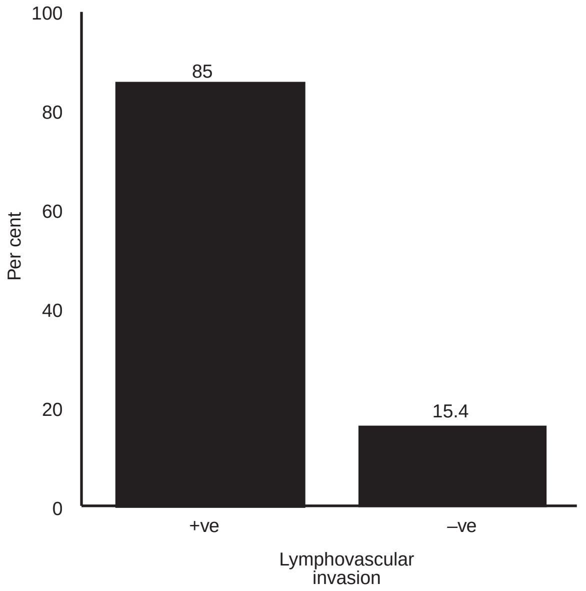

**Figure 4** Axillary lymph node positivity (%) in relation to lymphovascular invasion in the primary tumour

A strong correlation was found between the presence of lymphovascular invasion and axillary nodal metastases with 85% of tumours exhibiting this feature being associated with a positive axilla (Figure 4). Approximately 45% of patients with ER +ve/PgR +ve tumours had positive axillary nodes, but absence of one or both types of hormone receptors increased positivity by about 8–13% (Figure 5).

Multifactorial analyses showed that only 6.4% and 10.7% of patients with T1b and T1c tumours without lymphovascular invasion, respectively, had axillary nodal metastases, whereas for grade I T2 tumours without lymphovascular invasion the corresponding figure was 30.7%. These figures are lower than the corresponding figures in Figure 3 and Table 2, showing that the addition of histological grade and lymphovascular invasion to tumour size can improve further the accuracy with which these parameters predict axillary lymph node status.

### DISCUSSION

Over the last few years the surgical approach to breast cancer has become less aggressive and the majority of patients have breast conserving operations, with mastectomy being reserved for locally advanced or multifocal tumours. However, in most specialist units nearly all patients with invasive breast carcinoma undergo some form of axillary dissection ranging from axillary sampling to a full clearance. The importance of axillary lymph node status in determining prognosis in breast cancer and helping in the planning of adjuvant therapy is widely accepted. Moreover, axillary clearance contributes to the local control of the disease and reduces the incidence of axillary recurrence (Kjaergaard et al, 1985; Benson and Thorogood, 1986; Hayward and Caleffi, 1987). Harris and Osteen (1985) have reported improved survival in patients following axillary surgery, although most reports have shown that survival remains unchanged (Fisher et al, 1985).

The potential benefits of axillary dissection must be balanced against the well-known complications of this procedure (Benson and Thorogood, 1986; Gutman et al, 1990; Hoe et al, 1992), such as arm lymphoedema, sensory loss in the upper inner arm and restriction of shoulder movements, which although rare and usually short lived, can be very distressing to the patient. Moreover, routine axillary dissection is associated with increased hospital expense because of longer recovery time and stay in hospital as well as longer operating time.

Early treatment failure, manifesting as local or distant tumour recurrence, in node-negative patients has focused interest on some other prognostic indicators. Tumour size has been related to prognosis for many years (Fisher et al, 1969) and is an independent predictor of disease-free and overall survival for both lymph node-positive and negative patients (Crowe et al, 1992). Histological

© 1999 Cancer Research Campaign grade and lymphovascular invasion have also been shown to have significant prognostic implications in patients with breast cancer, more so in those with negative lymph nodes (Lauria et al, 1995; Roberti, 1997).

With the introduction of breast screening, the presentation of women with breast cancer has changed and early-stage tumours are now diagnosed with ever-increasing frequency. Routine axillary node clearance in all cases is, therefore, not justifiable and for some early cancers not useful. In this series, nearly 25% of patients had screen detected tumours, 55.4% had T1 tumours and 14.2% had tumours up to 1 cm in diameter. We feel that in the absence of screening these figures would have been smaller.

Approximately, 45% of the patients in this study had axillary lymph node metastases. Could the other 55% have been identified preoperatively so that axillary dissection could have been avoided? Our results confirm the direct relationship between primary tumour histopathological characteristics such as size, histological grade and lymphovascular invasion, and lymph node status. None of the patients with DCIS and T1a tumours had axillary metastases, but these were present in 16.3% and 35.7% of cases with T1b and T1c tumours respectively. Lymphovascular invasion was also a very important factor as 85% of tumours with this feature were associated with nodal metastases, as compared to only 15.4% of tumours without this feature. Similar figures have been reported in a recent study in which axillary metastases were present in 3% of patients with T1a tumours, however, the association between lymphovascular invasion and axillary nodal positivity was not as strong as in our study (Silverstein et al, 1994). The authors of this report suggested that axillary dissection is not necessary for patients with T1a tumours as the yield of positive lymph nodes is very small and only 1% of these patients will benefit from any subsequent chemotherapy. Combinations of various clinicopathological parameters can be more accurate predictors of axillary nodal positivity and Barth showed that 49% of patients with palpable, T1c, high nuclear grade tumours with lymphovascular invasion had axillary lymph node metastases, as compared to only 3% of those with T1a and T1b tumours and without the other three risk factors (Barth et al, 1997). Similarly, our study shows that the absence of lymphovascular invasion in T1b, T1c and grade I T2 tumours is associated with a 9.9%, 25% and 11.4% reduction in axillary node positivity respectively.

Based on the results presented and discussed here, we feel that patients with operable breast cancer should be divided into three categories: (a) those with DCIS and T1a tumours who do not require routine axillary lymph node dissection; (b) those with T1b, T1c and grade I T2 tumours in whom axillary dissection should be carried out selectively depending on lymphovascular invasion and other biological parameters including DNA ploidy, hormone receptors, growth factors and their receptors, and oncogenes; and (c) those with tumours greater than 2 cm in diameter or palpable lymph nodes, in whom axillary dissection (usually clearance) is a necessary intervention because of the high frequency of nodal metastases. Although it has been argued that in the latter group axillary dissection is not necessary because adjuvant treatment is usually given (Cady, 1997), irrespective of nodal status, we feel that axillary dissection improves local control of the disease and by determining the extent of nodal involvement, it helps decide which is the most appropriate chemotherapeutic regime.

The technique of sentinel lymph node biopsy first used in the treatment of penile cancer (Cabanas, 1977) and malignant melanoma (Morton et al, 1992), has recently been introduced in the management of patients with breast cancer (Krag et al, 1993; Giuliano et al 1994). Initial reports show high sensitivity and specificity in predicting axillary lymph node status (Albertini et al, 1996), with few false-negative results, most of them in tumours over 1.5 cm and those showing multifocality (Veronesi et al, 1997). We feel that this method could be very useful for all patients with tumours in category b, i.e. T1b, T1c and grade I T2 tumours. It can be carried out, if appropriate, on a day-surgery basis and depending on results, the patient will be selected for further surgery if necessary. Another advantage of this method is that it will permit a more detailed analysis of the one or two sentinel lymph nodes removed, by examining multiple sections and using immunohistochemistry. This intense histological examination will result in the detection of lymph node metastases, even micrometastases, missed by current techniques (Giuliano et al, 1995). Evidence suggests that the detection of micrometastases has prognostic significance and may help in the planning of adjuvant therapy (McGuckin et al, 1996; Dowlatshahi et al, 1997).

We are entering a new era in the management of the axilla in patients with breast cancer. Axillary dissection should be eliminated for DCIS and T1a tumours and performed selectively for patients with T1b, T1c and grade I T2 tumours. Primary tumour characteristics such as size, histological type and grade, lymphovascular invasion, and expression of growth factors and oncogenes will continue to provide valuable prognostic information and, because of their correlation with axillary lymph node status, should be used as criteria in selecting patients for axillary dissection. Moreover, sentinel lymph node biopsy will most probably establish itself as an accurate axillary staging procedure for the groups of patients discussed above.

Preoperative risk assessment is an important aspect of everyday surgical practice. The management of breast cancer is no exception and patients should be given a clear account of the benefits and risks of axillary dissection. We hope that the results presented here can be useful in the preoperative counselling of patients with breast cancer and contribute to the establishment of simple and reliable criteria which will simplify the selection process for axillary lymphadenectomy.

### ACKNOWLEDGEMENTS

We thank Emma Bennett for help in the collection and analysis of the data.

### REFERENCES

Albertini JJ, Lyman GH, Cox C, Yeatman T, Balducci L et al (1996) Lymphatic mapping and sentinel lymph node biopsy in the patient with breast cancer. *JAMA* **276**: 1818–1822

Avril N, Dose J, Janicke F, Ziegler S, Romer W, Weber W, Herz M, Nathrath W, Graeff H and Schwaiger M (1996) Assessment of axillary lymph node involvement in breast cancer patients with positron emission tomography using radiolabeled 2-(fluorine-18)-fluoro-2-deoxy-D-glucose. *J Natl Cancer Inst* **88**: 1204–1209

Barth A, Craig PH and Silverstein MJ (1997) Predictors of axillary lymph node metastases in patients with T1 breast carcinoma. *Cancer* **79**: 1918–1922

Benson EA and Thorogood J (1986) The effect of surgical technique on local recurrence rates following mastectomy. *Eur J Surg Oncol* **12**: 267–271

Bruneton JN, Caramella E, Hery M et al (1986) Axillary lymph node metastases in breast cancer: preoperative detection with US. *Radiology* **158**: 325–326

Cabanas RM (1977) An approach for the treatment of penile carcinoma. *Cancer* **39**: 456–466

Cabanes PA, Salmon RJ, Vilcoq JR, Durand JC, Fourquet A, Gautier C and Asselain B, for the breast carcinoma collaborative group of the Institut Curie (1992)

© 1999 Cancer Research Campaign 

Value of axillary dissection in addition to lumpectomy and radiotherapy in early breast cancer. *Lancet* **339**: 1245–1248
Cady B (1997) Use of primary breast carcinoma characteristics to predict lymph node metastases. *Cancer* **79**: 1856–1861
Crowe JP, Gordon NH, Shenk RR, Zollinger RM, Brumberg DJ and Shuck JM (1992) Primary tumour size: relevance to breast cancer survival. *Arch Surg* **127**: 910–916
Dowlatshahi K, Fan M, Snider HC and Habib FA (1997) Lymph node micrometastases from breast carcinoma. *Cancer* **80**: 1188–1197
Elston CW and Ellis IO (1991) Pathological prognostic factors in breast cancer. I. The value of histological grade in breast cancer: experience from a large study with long term follow up. *Histopathology* **19**: 403–410
Fentiman IS and Mansel RE (1991) The axilla: not a no-go zone. *Lancet* **337**: 221–223
Fisher B, Slack NH, Bross IDJ et al (1969) Cancer of the breast: size of neoplasm and prognosis. *Cancer* **24**: 1071–1080
Fisher B, Redmond C, Fisher ER, Bauer M, Wolmark N, Wickerham L et al (1985) Ten-year results of a randomised clinical trial comparing radical mastectomy and total mastectomy with or without radiation. *N Engl J Med* **312**: 674–681
Giuliano AE, Kirgan DM, Guenther JM and Morton DL (1994) Lymphatic mapping and sentinel lymphadenectomy for breast cancer. *Ann Surg* **220**: 391–401
Giuliano AE, Dale PS, Turner RR, Morton DL, Evans SW and Kranse DL (1995) Improved axillary staging of breast cancer with sentinel lymphadenectomy. *Ann Surg* **222**: 394–401
Greenall MJ (1995) Why I favour axillary node sampling in the management of breast cancer. *Eur J Surg Oncol* **21**: 2–5
Gutman H, Kersz T, Barzilai T et al (1990) Achievements of physical therapy in patients after modified radical mastectomy compared with quadrantectomy, axillary dissection, and radiation for carcinoma of the breast. *Arch Surg* **125**: 389–391
Harris JR and Osteen RT (1985) Patients with early breast cancer benefit from effective axillary treatment. *Breast Cancer Res Treat* **5**: 17–21
Hayward JL and Caleffi M (1987) The significance of local control in the primary treatment of breast cancer. *Arch Surg* **122**: 1244–1247
Hoe AL, Iven D, Royle GT and Taylor I (1992) Incidence of arm swelling following axillary clearance for breast cancer. *Br J Surg* **79**: 261–262
Kissin MW, Thompson EM, Price AB, Slavin G and Kark AE (1982) The inadequecy of axillary sampling in breast cancer. *Lancet* **i**: 1210–1211
Kjaergaard J, Blichert-Toft M, Anderson J et al (1985) Probability of false negative nodal status in conjunction with partial axillary dissection in breast cancer. *Br J Surg* **72**: 365–367
Krag DN, Weaver DL, Alex JC and Fairbank JT (1983) Surgical resection and radiolocalisation of the sentinel lymph node in breast cancer using a gamma probe. *Surg Oncol* **1993**: 335–339
Lam WW, Yang WT, Chan YL, Stewart IE, Metreweli C and King W (1996) Detection of axillary lymph node metestases in breast carcinoma by technetium-99m sestamibi breast scintigraphy, ultrasound and conventional mammography. *Eur J Nucl Med* **23**: 498–503
Lauria R, Perrone F, Carlomagno C, De-Laurentiis M, Morabito A, Gallo C, Varriale E, Pettinato G, Panico L, Petrella G et al (1995) The prognostic value of lymphatic and blood vessel invasion in operable breast cancer. *Cancer* **76**: 1772–1778
McGuckin MA, Cummings MC, Walsh MD, Hohn BG, Bennett IC and Wright RG (1996) Occult axillary node metastases in breast cancer: their detection and prognostic significance. *Br J Cancer* **73**: 88–95
McLean RG and Ege GN (1986) Prognostic value of axillary lymphoscintigraphy in breast carcinoma patients. *J Nucl Med* **27**: 1116–1124
Morton D, Wen D and Cochran A (1992) Management of early-stage melanoma by intraoperative lymphatic mapping and selective lymphadenectomy: an alternative to routine elective lymphadenectomy or 'watch and wait'. *Surg Oncol Clin N Am* **1**: 247–259
Roberti NE (1997) The role of histologic grading in the prognosis of patients with carcinoma of the breast. *Cancer* **80**: 1708–1716
Sacre RA (1986) Clinical evaluation of axillary lymph nodes compared to surgical and pathological findings. *Eur J Surg Oncol* **12**: 169–173
Silverstein MJ, Gierson ED, Waisman JR, Senofsky GM, Colburn WJ and Gamagami P (1994) Axillary lymph node dissection for T1a breast carcinoma. *Cancer* **73**: 664–667
Steele RJC, Forrest APM, Gibson T et al (1985) The efficacy of lower axillary sampling in obtaining lymph node status in breast cancer: a controlled randomised trial. *Br J Surg* **72**: 368–369
Tjandra JJ, Russell IS, Collins JP, Andrews JT, Lichtenstein M, Binns D and McKenzie IF (1989*a*) Immunolymphoscintigraphy for the detection of lymph node metastases from breast cancer. *Cancer Res* **49**: 1600–1608
Tjandra JJ, Sacks NP, Thompson CH, Leyden MJ, Stacker SA, Lichtenstein M, Russell IS, Collins JP, Andrews JT, Pietersz GA et al (1989*b*) The detection of axillary lymph node metastases from breast cancer by radiolabelled monoclonal antibodies: a prospective study. *Br J Cancer* **59**: 296–302
Veronesi U, Paganelli G, Galimberti V, Viale G, Zurrida S, Bedoni M, Costa A, de Cicco C, Geraghty JG, Luini A, Szcchini V and Veronesi P (1997) Sentinel-node biopsy to avoid axillary dissection in breast cancer with clinically negative lymph-nodes. *Lancet* **349**: 1864–1867

 *© 1999 Cancer Research Campaign*
